# Programs for the calculation of the spinodal decomposition growth rate and the spinodal gap in nanoparticles

**DOI:** 10.1016/j.dib.2017.10.038

**Published:** 2017-10-25

**Authors:** Evgeny Pogorelov, Julia Kundin

**Affiliations:** a Advanced Ceramics Group, University Bremen, D-28359 Bremen, Germany; b ICAMS, Ruhr-University Bochum, D-44801 Bochum, Germany

## Abstract

This data article contains the programs for the calculation of the spinodal decomposition growth rate and for the modeling of the spinodal gap and concentration profiles in nanoparticles which were used in our article (Pogorelov et al., 2017) [1]. The modeling is based on the mathematical model of spinodal phase decomposition with intercalation rate conditions on the boundaries (Singh et al., 2008) [2].

The maximal growth rate and the parameters of the concentration wave function can be evaluated for a fixed mean composition and intercalation rate. Furthermore, the maximal growth rate as a function of concentration and particle site can be evaluated for various intercalation rates.

**Specifications Table**

**Table:** 

Subject area	*Physics*
More specific subject area	*Spinodal decomposition*
Type of data	*Text file*
How data was acquired	*Python software, numpy open-source python library*
Data format	*Python language*
Experimental factors	*–*
Experimental features	*–*
Data source location	*Bremen, Germany*
Data accessibility	*Data is enclosed to this article*

**Value of the data**

• The programs are used to analyze of spinodal decomposition in nanoparticles to study the dependence of spinodal gap on the boundary reaction rate and the particle size.
• The codes can be useful for other researchers in the development of further programs for spinodal decomposition in nanoparticles with the intercalation effects.
• The programs are important for the design of stable batteries in energetic technology.

## 1 Data

### 1.1 Program “smax_sp_gap_color.py” for the calculation of the maximal growth rate distribution

The program is used for the calculation of the maximal growth rate as a function of the concentration, c0, and the particle size, L, for various intercalation rates, R. The maximal growth rate is plotted in a 3D color map. The plot also visualizes the spinodal gap.

########################

# smax_sp_gap_color.py #

########################

import numpy as np

from scipy.optimize import brentq

import matplotlib.pyplot as plt

from matplotlib import cm

from matplotlib.colors import LinearSegmentedColormap

#To help make good color distribution shifted from default one

def rescaled_colormap(cmap, left, right):

 left=float(left)

 right=float(right)

 cleft=left>0.

 cright=right<1.

 if left>=right: return cmap

 if not cleft: left=0.

 if not cright: right=1.

 cdict=(cmap._segmentdata).copy()

 for k, vs in cdict.iteritems():

  v=list(vs)

  if cleft: v.append((left, 0., 0.))

  if cright: v.append((right, 0., 0.))

  v.sort()

  vnew=[]; addpnts=False

  for i in xrange(len(v)):

   if v[i][0]==left:

   if left==0.: vc=v[i][2]

   else: vc=v[i−1][2]+(v[i][0]−v[i−1][0])/(v[i+1][0]−v[i−1][0])*(v[i+1][1]−v[i−1][2])

  v[i]=(left, vc, vc)

  addpnts=True

 if v[i][0]==right:

  if right==1.: vc=v[i][1]

  else: vc=v[i−1][2]+(v[i][0]−v[i−1][0])/(v[i+1][0]−v[i−1][0])*(v[i+1][1]−v[i−1][2])

  v[i]=(right, vc, vc)

  vnew.append((1., vc, vc))

  addpnts=False

 if addpnts: vnew.append(((v[i][0]-left)/(right-left), v[i][1], v[i][2]))

cdict[k]=vnew

return LinearSegmentedColormap('my_cmap', cdict, cmap.N)

kT=1.

a=5*kT

lam=4.

D=1.

M=D/kT

K=kT*lam**2

rho_s=1.

rho=rho_s/lam

cmin=(1-np.sqrt(1–2*kT/a))/2

cmax=(1+np.sqrt(1–2*kT/a))/2

print 'cmin={0}, cmax={1}, diff={2}'.format(cmin, cmax, cmax-cmin)

R_diml=5. # intercalation rate

R_ins=D*R_diml/lam*rho/rho_s

Rb=D*R_diml/lam/kT*rho

l=np.linspace(0.01, 5, 400)

l_sh=l.shape[0]

L=l*lam

cstep=0.001 ns=200

bmatr=np.empty((ns, 4, 4), np.float)

cmatr=np.empty((4, 4), np.float)

dtr=np.empty(l_sh)

def res0(c):

 #nmax=int(lam*l[-1]/np.pi/np.sqrt(K/(2*a-4*kT)))

 nmax=3

 print 'nmax={0}'.format(nmax)

 n=np.arange(nmax)+1

 det2=1–4*kT/(2*a-K*(n*np.pi/L[-1])**2)

 c0=0.5-np.sqrt(det2)/2

 c1=0.5+np.sqrt(det2)/2

 ans=[]

 for i in xrange(nmax):

  c_cur=c[np.where((c>c0[i])&(c<c1[i]))]

  l_cur=n[i]*np.pi*np.sqrt(K/(2*a-kT/c_cur/(1-c_cur)))/lam

  ans.extend([l_cur, c_cur, 'k--'])

 return ans

def res_max(x, c):

 d2g=−2*a+kT/c/(1-c)

 s=c*M*d2g**2/4/K

 k=np.sqrt(−d2g/2/K)

 X=x*lam

 sl=−12*np.sin(k*X)*np.cos(k*X)*np.sqrt(−2*K*d2g)*Rb*rho*c*M

 sl+=4*Rb*d2g*rho*c*M*X-4*d2g*(rho*c*M)**2+4*d2g*(rho*c*M*np.cos(k*X))**2

 sl+=−d2g*(Rb*X)**2–18*Rb**2*K+18*K*(Rb*np.cos(k*X))**2

 if Rb==0: sl=np.sin(k*X)

 if sl>0: return 1

 else: return -1

def res_maxv(c):

 d2g=−2*a+kT/c/(1-c)

 s=c*M*d2g**2/4/K

 k=np.sqrt(-d2g/2/K)

 sl=−12*np.sin(k*L)*np.cos(k*L)*np.sqrt(-2*K*d2g)*Rb*rho*c*M

 sl+=4*Rb*d2g*rho*c*M*L-4*d2g*(rho*c*M)**2+4*d2g*(rho*c*M*np.cos(k*L))**2

 sl+=−d2g*(Rb*L)**2–18*Rb**2*K+18*K*(Rb*np.cos(k*L))**2

 if Rb==0: sl=np.sin(k*L)

 dtr.setfield(np.where(sl>0, 1, −1), np.float)

 answer=[]

 ddtr=np.diff(dtr)

 ind_left=np.where(ddtr<>0)[0]

 ind_right=ind_left+1

 for i in xrange(ind_left.shape[0]):

  answer.append(brentq(res_max, l[ind_left[i]], l[ind_right[i]], xtol=cstep/10, args=(c,)))

 return answer

def det_s(s,c,x):

 d2g=−2*a+kT/(c*(1−c))

 d2=d2g**2–4*K*s/(M*c)

 kmin=(−d2g-np.sqrt(d2))/(2*K)

 kmax=(−d2g+np.sqrt(d2))/(2*K)

 kmin=np.sqrt(kmin); kmax=np.sqrt(kmax)

 cmatr[0, 0]=kmin

 cmatr[0, 1]=0.

 cmatr[0, 2]=kmax

 cmatr[0, 3]=0.

 cmatr[1, 0]=kmin*np.cos(kmin*x)

 cmatr[1, 1]=−kmin*np.sin(kmin*x)

 cmatr[1, 2]=kmax*np.cos(kmax*x)

 cmatr[1, 3]=−kmax*np.sin(kmax*x)

 cmatr[2, 0]=−rho*c*M*kmin*(K*kmin**2+d2g)

 cmatr[2, 1]=Rb*(K*kmin**2+d2g)

 cmatr[2, 2]=−rho*c*M*kmax*(K*kmax**2+d2g)

 cmatr[2, 3]=Rb*(K*kmax**2+d2g)

 cmatr[3, 0]=(K*kmin**2+d2g)*(rho*M*c*np.cos(kmin*x)*kmin+Rb*np.sin(kmin*x))

 cmatr[3, 1]=-(K*kmin**2+d2g)*(rho*M*c*np.sin(kmin*x)*kmin-Rb*np.cos(kmin*x))

 cmatr[3, 2]=(K*kmax**2+d2g)*(rho*M*c*np.cos(kmax*x)*kmax+Rb*np.sin(kmax*x))

 cmatr[3, 3]=-(K*kmax**2+d2g)*(rho*M*c*np.sin(kmax*x)*kmax-Rb*np.cos(kmax*x))

 return np.linalg.slogdet(cmatr)[0]

def smaxf(c, x):

 d2g=−2*a+kT/(c*(1−c))

 smax=c*M*d2g**2/(4*K)

 s=np.linspace(smax/100000, smax*(1-1./100000), ns)

 d2=(c*M*d2g)**2–4*c*M*K*s

 kmin=(−c*M*d2g-np.sqrt(d2))/(2*c*M*K)

 kmax=(−c*M*d2g+np.sqrt(d2))/(2*c*M*K)

 kmin=np.sqrt(kmin); kmax=np.sqrt(kmax)

 bmatr[:, 0, 0]=kmin

 bmatr[:, 0, 1]=np.zeros(kmin.shape)

 bmatr[:, 0, 2]=kmax

 bmatr[:, 0, 3]=np.zeros(kmax.shape)

 bmatr[:, 1, 0]=kmin*np.cos(kmin*x)

 bmatr[:, 1, 1]=−kmin*np.sin(kmin*x)

 bmatr[:, 1, 2]=kmax*np.cos(kmax*x)

 bmatr[:, 1, 3]=−kmax*np.sin(kmax*x)

bmatr[:, 2, 0]=−rho*c*M*kmin*(K*kmin**2+d2g)

 bmatr[:, 2, 1]=Rb*(K*kmin**2+d2g)

 bmatr[:, 2, 2]=−rho*c*M*kmax*(K*kmax**2+d2g)

 bmatr[:, 2, 3]=Rb*(K*kmax**2+d2g)

 bmatr[:, 3, 0]=(K*kmin**2+d2g)*(rho*(M*c)*np.cos(kmin*x)*kmin+Rb*np.sin(kmin*x))

 bmatr[:, 3, 1]=−(K*kmin**2+d2g)*(rho*(M*c)*np.sin(kmin*x)*kmin-Rb*np.cos(kmin*x))

 bmatr[:, 3, 2]=(K*kmax**2+d2g)*(rho*(M*c)*np.cos(kmax*x)*kmax+Rb*np.sin(kmax*x))

 bmatr[:, 3, 3]=−(K*kmax**2+d2g)*(rho*(M*c)*np.sin(kmax*x)*kmax-Rb*np.cos(kmax*x))

 for i in xrange(ns):

  sl=np.linalg.slogdet(bmatr[i,:,:])

  dtr[i]=sl[0]

 s_sol=−0.001*D/lam**2

 for i in xrange(ns-2, −1, −1):

  if abs(dtr[i]+dtr[i+1])<>2:

   s_sol=brentq(det_s, s[i], s[i+1], args=(c, x), rtol=1e-5)

   break

 return s_sol*lam**2/D

c=np.arange(cmin+0.0005, cmax-0.0005, cstep)

xs=[]

ys=[]

for c0 in c:

 roots=res_maxv(c0)

 for root in roots:

  xs.append(root)

  ys.append(c0)

csh=c.shape[0]; lsh=l.shape[0]

stbl=np.empty((csh, lsh), np.float)

for i in xrange(lsh):

 for j in xrange(csh):

  stbl[j, i]=smaxf(c[j], L[i])

 print l[i], np.max(stbl[:, i])

fig=plt.figure(0)

ax=fig.add_subplot(111)

cax=ax.imshow(stbl, extent=[np.min(l), np.max(l), np.min(c), np.max(c)], aspect='auto', origin='lower', cmap=rescaled_colormap(cm.hot_r, 0.25, 2.), vmin=0., vmax=np.max(stbl), interpolation='sinc')

cax.cmap.set_under('white')

ax.autoscale(False)

cbar=fig.colorbar(cax, orientation='horizontal')

cbar.set_label(r'$/bar{s}$', fontsize=20)

cbar.set_clim(0, np.max(stbl))

ax.scatter(xs, ys, s=2.0)

ax.plot(*res0(c), linewidth=2.)

ax.set_xlabel(r'$L//lambda$', fontsize=20)

ax.set_ylabel(r'$c_0$', fontsize=20)

fn=plt.figure(1)

axn=fn.add_subplot(111)

axn.plot(l, stbl[c.shape[0]/2,:])

axn.set_xlabel(r'$L//lambda$', fontsize=20)

axn.set_ylabel(r'$/max(/bar{s})$')

plt.show()

##########################

# end of “smax_sp_gap_color.py” #

##########################

### 1.2 Program “smax_section.py” for the calculation of the maximal growth rate and model parameters for a fixed concentration

The program is used for the calculation of the maximal growth rate, s max, and corresponding model parameters: the particle size, L, wave numbers, k_i_, amplitude coefficients, A_i_. The input parameters for the calculation are the intercalation rate, R, and a mean concentration, c0. The output is written in the output file “para_set.dat”.

########################

# smax_section.py #

########################

import numpy as np

from scipy.optimize import brentq

import matplotlib.pyplot as plt

kT=1.

a=5*kT

lam=4.

D=1.

M=D/kT

K=kT*lam**2

rho_s=1.

rho=1.

cmin=(1−np.sqrt(1–2*kT/a))/2

cmax=(1+np.sqrt(1–2*kT/a))/2

print 'cmin={0}, cmax={1}, diff={2}'.format(cmin, cmax, cmax-cmin)

R_diml=5. # intercalation rate

Rb=R_diml*M/lam

R_ins=D*R_diml/lam*rho/rho_s

Rb=D*R_diml/lam/kT*rho

cmatr=np.empty((4, 4), np.float)

ns=100

bmatr=np.empty((ns, 4, 4), np.float)

dtr=np.empty(ns)

def det_s(s,c,x):

 d2g=−2*a+kT/(c*(1-c))

 d2=d2g**2–4*K*s/(M*c)

 kmin=(−d2g-np.sqrt(d2))/(2*K)

 kmax=(−d2g+np.sqrt(d2))/(2*K)

 kmin=np.sqrt(kmin); kmax=np.sqrt(kmax)

 cmatr[0, 0]=kmin

 cmatr[0, 1]=0.

 cmatr[0, 2]=kmax

 cmatr[0, 3]=0.

 cmatr[1, 0]=kmin*np.cos(kmin*x)

 cmatr[1, 1]=−kmin*np.sin(kmin*x)

 cmatr[1, 2]=kmax*np.cos(kmax*x)

 cmatr[1, 3]=−kmax*np.sin(kmax*x)

 cmatr[2, 0]=−rho*c*M*kmin*(K*kmin**2+d2g)

 cmatr[2, 1]=Rb*(K*kmin**2+d2g)

 cmatr[2, 2]=−rho*c*M*kmax*(K*kmax**2+d2g)

 cmatr[2, 3]=Rb*(K*kmax**2+d2g)

 cmatr[3, 0]=(K*kmin**2+d2g)*(rho*M*c*np.cos(kmin*x)*kmin+Rb*np.sin(kmin*x))

 cmatr[3, 1]=-(K*kmin**2+d2g)*(rho*M*c*np.sin(kmin*x)*kmin-Rb*np.cos(kmin*x))

 cmatr[3, 2]=(K*kmax**2+d2g)*(rho*M*c*np.cos(kmax*x)*kmax+Rb*np.sin(kmax*x))

 cmatr[3, 3]=-(K*kmax**2+d2g)*(rho*M*c*np.sin(kmax*x)*kmax-Rb*np.cos(kmax*x))

 return np.linalg.slogdet(cmatr)[0]

def get_A(s, c, x):

 d2g=−2*a+kT/c/(1-c)

 kmin=(−d2g - np.sqrt((d2g)**2–4*K*s/(M*c)))/(2*K)

 kmax=(−d2g+np.sqrt((d2g)**2–4*K*s/(M*c)))/(2*K)

 kmin=np.sqrt(kmin); kmax=np.sqrt(kmax)

 cmatr=np.empty((4, 4), np.float)

 cmatr[0, 0]=kmin

 cmatr[0, 1]=0.0

 cmatr[0, 2]=kmax

 cmatr[0, 3]=0.0

 cmatr[1, 0]=kmin*np.cos(kmin*x)

 cmatr[1, 1]=−kmin*np.sin(kmin*x)

 cmatr[1, 2]=kmax*np.cos(kmax*x)

 cmatr[1, 3]=−kmax*np.sin(kmax*x)

 cmatr[2, 0]=−rho*c*M*kmin*(K*kmin**2+d2g)

 cmatr[2, 1]=Rb*(K*kmin**2+d2g)

 cmatr[2, 2]=−rho*c*M*kmax*(K*kmax**2+d2g)

 cmatr[2, 3]=Rb*(K*kmax**2+d2g)

 cmatr[3, 0]=(K*kmin**2+d2g)*(rho*M*c*np.cos(kmin*x)*kmin+Rb*np.sin(kmin*x))

 cmatr[3, 1]=-(K*kmin**2+d2g)*(rho*M*c*np.sin(kmin*x)*kmin-Rb*np.cos(kmin*x))

 cmatr[3, 2]=(K*kmax**2+d2g)*(rho*M*c*np.cos(kmax*x)*kmax+Rb*np.sin(kmax*x))

 cmatr[3, 3]=−(K*kmax**2+d2g)*(rho*M*c*np.sin(kmax*x)*kmax-Rb*np.cos(kmax*x))

 U, singular, V=np.linalg.svd(cmatr)

 A=V[3,:]

 return lam*kmin, lam*kmax, s*lam**2/D, A[0], A[1], A[2], A[3], x/lam, c

def smaxf(c, x):

 d2g=−2*a+kT/(c*(1−c))

 smax=c*M*d2g**2/(4*K)

 s=np.linspace(smax/ns/100, smax*(1-1./ns/100), ns)

 d2=(c*M*d2g)**2–4*c*M*K*s

 kmin=(−c*M*d2g-np.sqrt(d2))/(2*c*M*K)

 kmax=(−c*M*d2g+np.sqrt(d2))/(2*c*M*K)

 kmin=np.sqrt(kmin); kmax=np.sqrt(kmax)

 bmatr[:, 0, 0]=kmin

 bmatr[:, 0, 1]=np.zeros(kmin.shape)

 bmatr[:, 0, 2]=kmax

 bmatr[:, 0, 3]=np.zeros(kmax.shape)

 bmatr[:, 1, 0]=kmin*np.cos(kmin*x)

 bmatr[:, 1, 1]=−kmin*np.sin(kmin*x)

 bmatr[:, 1, 2]=kmax*np.cos(kmax*x)

 bmatr[:, 1, 3]=−kmax*np.sin(kmax*x)

 bmatr[:, 2, 0]=−rho*c*M*kmin*(K*kmin**2+d2g)

 bmatr[:, 2, 1]=Rb*(K*kmin**2+d2g)

 bmatr[:, 2, 2]=−rho*c*M*kmax*(K*kmax**2+d2g)

 bmatr[:, 2, 3]=Rb*(K*kmax**2+d2g)

 bmatr[:, 3, 0]=(K*kmin**2+d2g)*(rho*(M*c)*np.cos(kmin*x)*kmin+Rb*np.sin(kmin*x))

 bmatr[:, 3, 1]=−(K*kmin**2+d2g)*(rho*(M*c)*np.sin(kmin*x)*kmin-Rb*np.cos(kmin*x))

 bmatr[:, 3, 2]=(K*kmax**2+d2g)*(rho*(M*c)*np.cos(kmax*x)*kmax+Rb*np.sin(kmax*x))

 bmatr[:, 3, 3]=−(K*kmax**2+d2g)*(rho*(M*c)*np.sin(kmax*x)*kmax-Rb*np.cos(kmax*x))

 for i in xrange(ns):

  sl=np.linalg.slogdet(bmatr[i,:,:])

  dtr[i]=sl[0]

 s_sol=−0.001

 for i in xrange(ns-2, −1, −1):

 if abs(dtr[i]+dtr[i+1])<>2:

  s_sol=brentq(det_s, s[i], s[i+1], args=(c, x), rtol=1e-5)

  P=get_A(s_sol, c, x)

  out_string='{0} {1} {2} {3} {4} {5} {6} {7} {8}'.format(*P)+'/n'

  outfile.write(out_string)

  break

 return s_sol*lam**2/D

outfile=open('para_set.dat', 'w')

outfile.write('#1. lam_k1 ' + '2. lam_k2 ' + '3. s_dl ' + '4. A1 ' + '5. A2 '+ '6. A3 '+ '7. A4 ' + '8. l ' + ' 9. c_0 '+'/n')

c_step=1

l_step=1000

c=np.linspace(cmin+0.0005, cmax-0.0005, c_step)

c[0]=0.5

l=np.linspace(0.1, 5.0, l_step)

L=lam*l

stbl=np.empty((l_step, c_step), np.float)

for i in xrange(l_step):

 print L[i]

 for j in xrange(c_step):

  stbl[i, j]=smaxf(c[j], L[i])

outfile.close()

plt.imshow(stbl, extent=[np.min(l), np.max(l), np.min(c), np.max(c)], aspect='auto')

plt.show()

#####################

# end of “smax_section.py” #

#####################

### 1.3 Program “s_relative.py” for the validation of the relative growth rate

The program is used for the calculation of the relative growth rate, s, of spinodal decomposition for an initial noise function. The program allows to simulate the concentration profile function for various intercalation rates and particle size. The calculated relative growth rate can be compared to analytical predictions. The input data are taken from the input file “para_set.dat” which contains parameters for the noise function. The output file “output.txt” contains the relative growth rate. The concentration, c1, is plotted as a function of the coordinate, x. Then it is saved in the file “ampl.eps”.

#####################

# s_relative.py #

#####################

import numpy as np

import matplotlib.pyplot as plt

import sys

#Read of parameters

lamkmin=[]; lamkmax=[]; sdl=[]

A1=[]; A2=[]; A3=[]; A4=[]

Length=[]; C_0=[]

infile=open('para_set.dat','r')

infile.readline()

lines=infile.readlines()

for line in lines:

 ln=line.split()

 lamkmin.append(float(ln[0]))'ampl.eps'

 lamkmax.append(float(ln[1]))

 sdl.append(float(ln[2]))

 A1.append(float(ln[3]))

 A2.append(float(ln[4]))

 A3.append(float(ln[5]))

 A4.append(float(ln[6]))

 Length.append(float(ln[7]))

 C_0.append(float(ln[8]))

infile.close()

nx=100 # number of grid points in x direction

kT=1.

a=5*kT

lam=4.

D=1.

M=D/kT

K=kT*lam**2

rhos=1.

rho=1.

R_diml=5. # intercalation rate

R_ins=D*R_diml/lam*rho/rhos

Rb=R_ins

outfile=open('output.txt', 'w')

output_to_file='1.n 2.s_dl 3.l 4.c 5.err'

outfile.write(output_to_file)

num_of_lines=len(A1)

plt.ion()

fig=plt.figure()

max_rel_error=0.0

for n in xrange(num_of_lines):

 L=Length[n]*lam

 lx=L

 kmax=lamkmax[n]/lam

 kmin=lamkmin[n]/lam

 c_init=C_0[n]

 mu_ext=a*(1–2*c_init)+kT*(np.log(c_init)-np.log(1-c_init))

 dg2_c0=−2*a+kT/c_init/(1−c_init)

 dx=L/nx; dx2=dx**2; dx3=dx**3; dx4=dx**4

 x=np.linspace(−2*dx, L+2*dx, nx+5)

 dt=dx4*0.001

 sh=x.shape

 #initialization

 c=A1[n]* np.sin(kmin*x)+A2[n]* np.cos(kmin*x)+A3[n]* np.sin(kmax*x)+A4[n]* np.cos(kmax*x)

# initial noise function

 c=c/max(abs(np.max(c)), abs(np.min(c)))

 c[1]=c[3]; c[−2]=c[−4]

 mu2=c[2]*dg2_c0-K*(c[1]+c[3]−2*c[2])/dx2

 mum3=c[−3]*dg2_c0-K*(c[−4]+c[−2]−2*c[−3])/dx2

 c[0]=c[4]+rhos*Rb*(mu2/kT)*2*dx3/rho/c_init/M/K

 c[−1]=c[−5]+rhos*Rb*(mum3/kT)*2*dx3/rho/c_init/M/K

 av_c=np.sum(np.abs(c[2:−2]))*dx/nx

 ax=fig.add_subplot(111)

 ln,=ax.plot(x/lam, c)

 ax.set_xlim(−2*dx/lam, (lx+2*dx)/lam)

 ax.set_ylim(np.min(c)-0.1*(np.max(c)-np.min(c)), np.max(c)+0.1*(np.max(c)-np.min(c)))

 ax.set_xlabel(r'$/bar{x}$', fontsize=20)

 ax.set_ylabel(r'$c_1(/bar{x},/bar{t})//exp(/bar{s}/bar{t})$', fontsize=20)

 ax.grid()

 t=0

 i=0

 stop=False

 av_c_old=av_c

 sdln=sdl[n]

 stop=False

 s_dl_old=0.

 while not stop:

  c2=np.roll(c, −2); c1=np.roll(c, −1); cm1=np.roll(c, 1); cm2=np.roll(c, 2)

  c+=dt*M*c_init*((c1−2*c+cm1)*dg2_c0/dx2−K*(c2−4*c1+6*c−4*cm1+cm2)/dx4)

  c[1]=c[3]; c[−2]=c[−4]

  mu2=c[2]*dg2_c0−K*(c[1]+c[3]−2*c[2])/dx2

  mum3=c[−3]*dg2_c0-K*(c[−4]+c[−2]−2*c[−3])/dx2

  c[0]=c[4]+rhos*Rb*(mu2/kT)*2*dx3/rho/c_init/M/K

  c[−1]=c[−5]+rhos*Rb*(mum3/kT)*2*dx3/rho/c_init/M/K

  # intermediate output

  if i%1000==0:

   av_c=np.sum(np.abs(c[2:-2]))*dx/nx

   ln.set_ydata(c)

   plt.draw()

   plt.savefig('ampl.eps')

   sys.exit()

   s_dl=(np.log(av_c)-np.log(av_c_old))*lam**2/D/1000/dt

   stop=abs(s_dl-s_dl_old)<1e-7

   s_dl_old=s_dl

   av_c_old=av_c

  # Time update

  t+=dt

  i+=1

 rel_error=(s_dl-sdl[n])/sdl[n]*100

 if max_rel_error<abs(rel_error): max_rel_error=abs(rel_error)

 output='{0} s={1:.6f}, l={2:.6f}, c={3:.6f}, er&max er {4:.6f} %, {5:.6f} %'.format(n, sdl[n], Length[n], C_0[n], rel_error, max_rel_error)

 output_to_file='{0} {1} {2} {3} {4}/n'.format(n, sdl[n], Length[n], C_0[n], rel_error)

 print output

 outfile.write(output_to_file)

 ax.cla()

outfile.close()

#########################

# end of “ s_relative.py “ #

#########################
